# Blood donation practices, processing and utilisation of blood components in government tertiary hospitals in Nigeria: a multicentre cooperative study

**DOI:** 10.1093/inthealth/ihad105

**Published:** 2023-11-13

**Authors:** Garba Umar, Ibrahim Abdulqadir, Ngozi Ugwu, Titilope Adeyemo, Nabila Yau, Abdulazziz Hassan, John Olaniyi, Abubakar Musa, Sharafa Abubakar, Muhammad Ndakotsu, Jasini James, Chika Uche, Awwal Musa, Chikadibia Ukoma, Benedict Nwogoh, Ekaete David, Angela Ugwu, Chizoba Nwankwo, Olaitan Omokanye, Aisha Abba, Temilola Owojuyigbe, Mujtabba Isyaku, Esther Obi, Ezra Jatau, Timothy Ekwere, Rashidat Oladosu-Olayiwola, Hezekiah Isah, Sirajo Diggi, Alexander Nwannadi, Saleh Yuguda, Obinna Iheanacho, Hadiza Tikau, Ibijola Adeleke, Mabel Ekanem, Anazoeze Madu, Augustina Ikusemoro, Celestine Chukwu, Amal Galadanci, Okon Bassey, Theresa Otu, Obineche Agwu, Patrick Osho, Aisha Gwarzo, Sadiya Hassan, Adepoju Majeed, Anas Umar, Habib Abubakar, Mohamed Gimba, Michael Ugbor, Abdulmalik Ali, Clara Ajuba

**Affiliations:** Department of Haematology, Federal Medical Center, Birnin Kebbi, Kebbi State, Nigeria; Department of Haematology and Blood Transfusion, Usmanu Danfodiyo University Teaching Hospital, Sokoto, Nigeria; Department of Haematology and Immunology, Ebonyi State University, Abakaliki, Nigeria; Department of Haematology, University of Lagos, Lagos, Nigeria; Department of Haematology, College of Health Sciences, Federal University, Dutse, Jigawa State, Nigeria; Department of Haematology, Ahmadu Bello University Teaching Hospital, Zaria, Nigeria; Department of Haematology, University College Hospital, Ibadan, Nigeria; Department of Haematology, Federal Medical Center, Yola, Nigeria; Department of Haematology, Federal Medical Center, Yola, Nigeria; Department of Haematology, Federal Medical Center, Yola, Nigeria; Department of Haematology, Federal Medical Center, Yola, Nigeria; Department of Haematology, Abia State University Teaching Hospital, Aba Abai State, Nigeria; Department of Haematology, Murtala Muhammad Specialist Hospital, Kano, Nigeria; Department of Haematology, Federal Medical Center, , Keffi, Nassarawa State, Nigeria; Department of Haematology, University of Benin Teaching Hospital, Benin City, Nigeria; Department of Haematology, Natioanl Hospital, Abuja, Nigeria; Department of Haematology, University of Nigeria Teaching Hospital, Ituku-Ozalla, Enugu, Nigeria; Department of Haematology, Asokoro District Hospital, Abuja, Nigeria; Department of Haematology, University of Ilorin Teaching Hospital, Ilorin, Kwara State, Nigeria; Department of Haematology, University of Maiduguri Teaching Hospital, Bornu State, Nigeria; Department of Haematology, Obafemi Awolowo University Teaching Hospital Complex, Ile-Ife, Osun State, Nigeria; Department of Haematology, Federal Medical Center, Yenagoa, Bayelsa State, Nigeria; Department of Haematology, Federal Medical Center, Yenagoa, Bayelsa State, Nigeria; Department of Haematology, Jos University Teaching Hospital, Jos, Plateau State, Nigeria; Department of Haematology, University of Uyo Teaching Hospital, Uyo, Akwa Ibom State, Nigeria; Department of Haematology, Federal Medical Center, Abeokuta, Nigeria; Department of Haematology, University of Abuja Teaching Hospital, Abuja, Nigeria; Department of Haematology, Sir Yahaya Memorial Hospital, Birnin Kebbi, Kebbi State, Nigeria; Department of Haematology, Benue State University Teaching Hospital, Makurdi, Benue State, Nigeria; Department of Haematology, Federal Teaching Hospital, Gombe, Nigeria; Department of Haematology, University of Calabar, Cross River State, Nigeria; Department of Haematology, Federal Medical Center, Nguru, Yobe State, Nigeria; Department of Haematology, Federal Teaching Hospital, Ido Ekiti, Nigeria; Department of Haematology, University of Uyo Teaching Hospital, Uyo, Akwa Ibom State, Nigeria; Department of Haematology, University of Nigeria Teaching Hospital, Ituku-Ozalla, Enugu, Nigeria; Department of Haematology, Delta State University Teaching Hospital, Oghara, Delta State, Nigeria; Department of Haematology, National Neuropsychiatric hospital, Benin City, Edo State, Nigeria; Department of Haematology, National Neuropsychiatric hospital, Benin City, Edo State, Nigeria; Department of Haematology, University of Calabar Teaching Hospital, Calabar, Cross River State, Nigeria; Department of Haematology, University of Abuja Teaching Hospital, Abuja, Nigeria; Department of Haematology, Federal Teaching Hospital, Umuahia, Abia State, Nigeria; Department of Haematology, University of Medical Sciences Teaching Hospital, Ondo State, Nigeria; Department of Haematology, National Neuropsychiatric hospital, Benin City, Edo State, Nigeria; National Eye Centre, Kaduna, Nigeria; Department of Haematology, Federal Medical Center, Gusau, Nigeria; General Outpatient Clinic, Federal Medical Center, Birnin-Kudu, Nigeria; Department of Haematology, Rasheed Shekoni Specialist Hospital, Dutse, Jigawa State, Nigeria; Federal Neuropsychiatric Hospital, Kware, Sokoto State, Nigeria; Federal Neuropsychiatric Hospital, Kaduna, Nigeria; Department of Haematology, Muhammad Abdullahi Wase Specialist Hospital, Kano, Nigeria; Department of Haematology, Nnamdi Azikiwe University, Nnewi, Anambra State, Nigeria

**Keywords:** blood, blood component, blood donation, hepatitis, hospital, Nigeria

## Abstract

**Background:**

Timely access to safe blood and blood components is still a challenge in Nigeria. This study aimed to determine blood donation practices, processing and utilization of blood components across government tertiary hospitals (THs) in Nigeria.

**Methods:**

This was a descriptive cross-sectional study done in Nigeria in June–July 2020. Data were analysed with SPSS version 21.0.

**Results:**

Data were collected from 50 THs. The majority (68%) of the THs lack facilities for blood component preparation and only 18% and 32% provide cryoprecipitate and platelet concentrate, respectively. Whole blood was most commonly requested (57.04%). All facilities tested blood for HIV, HBV and HCV, but the majority (23 [46%]) employed rapid screening tests alone and nucleic acid testing was not available in any hospitals. The manual method was the most common method of compatibility testing in 90% (45/50) and none of the THs routinely perform extended red cell typing. The average time to process routine, emergency and uncross-matched requests were a mean of 109.58±79.76 min (range 45.00–360.00), 41.62±25.23 (10.00–240.00) and 11.09±4.92 (2.00–20.00), respectively.

**Conclusion:**

Facilities for blood component preparation were not widely available. Concerned government authorities should provide facilities for blood component preparation.

## Introduction

Blood and its component and products are necessary to prevent morbidity and mortality, especially in developing countries where reasonable alternatives are not readily available.^1–4^ For healthcare facilities to harness the benefit of blood in patient care, prompt availability and judicious use of safe blood must be ascertained, requiring the right donor and standard processing of blood.^5^ This will minimize complications, shorten delays and avoid blood wastage, especially when national blood transfusion services are not capable of meeting the demand for blood products and hospitals improve their capacities in terms of compatibility testing and transfusion transmissible infection (TTI) screening.^6–8^ In Nigeria, the National Blood Transfusion Commission (NBTC) is yet to provide the desired support to individual hospitals to provide blood component therapy to citizens. This implies a tasking challenge to individual healthcare facilities providing blood therapy to ensure self-sufficiency in blood processing and availability.^9–13^ The aim of this study was to determine the blood donation practices, processing of blood components and utilisation across all government tertiary hospitals (THs) in Nigeria.

## Methods

A descriptive cross-sectional study involving government-owned THs in Nigeria was conducted using a web-based (data was collected with electroiic google form) electronic questionnaire of 80 questions to collect information on hospitals, blood transfusion infrastructure, equipment and personnel, donor recruitment, blood donation and requisition, screening of TTIs, blood component preparation, blood security, inventory, records and blood transfusion management systems from June to July 2020. Data were collected from a haematologist in each centre, except in case of none available, where data were collected from another physician with an interest in blood transfusion or a medical laboratory scientist. Responses were collated directly by the research server into an Excel spreadsheet (Microsoft, Redmond, WA, USA). Data were imported into and analysed using SPSS version 21 (IBM, Armonk, NY, USA). Descriptive statistics were used to compute percentages and proportions, minimum, maximum, mean and standard deviation (SD).

Hospitals with incomplete responses or whose questionnaire was not received within the study period were excluded from the analysis.

## Results

**Figure 1.**
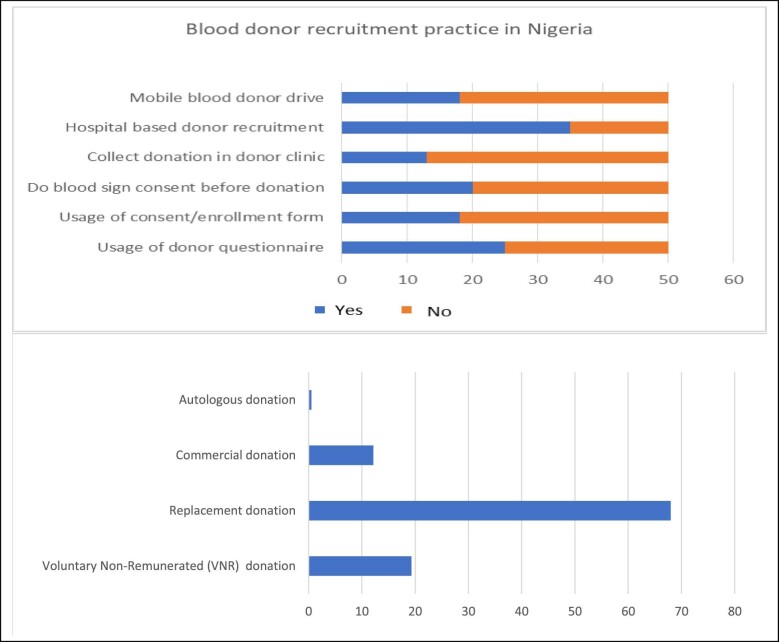
Donor recruitment in hospital-based transfusion in Nigeria.

**Figure 2.**
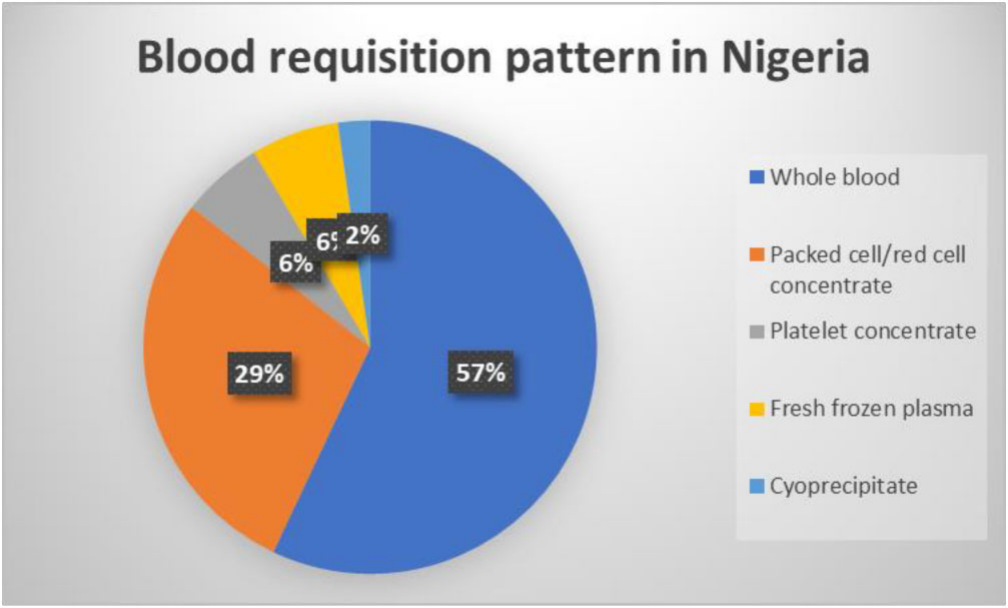
Blood component requisition pattern in hospital-based transfusion in Nigeria.

Fifty government THs of 103 met the inclusion criteria and were included in the study. Use of a donor questionnaire and enrolment form was practiced by 50% (25/50) and 36.0% (18/50), respectively, while 36.0% (18/50) had a donor clinic with a mobile donor drive to reach donors outside the hospital (Figure 1). Family replacement donors constitute the majority (68.0%) of blood donors, followed by voluntary non-remunerated blood donors (19.3%), commercial donors (12.2%) and autologous donors (0.3%) (Figure 1). Of the autologous donors, 63.13%, 26.74% and 10.13% were pre-deposit, preoperative haemodilution and intra-operative salvage, respectively. All hospitals screened blood for human immunodeficiency virus (HIV), hepatitis B and C viruses (HBV and HCV), but the majority (23 [46.0%]) employed a rapid screening test alone and nucleic acid testing was not available in any hospitals (Table 1). The majority of the hospitals (45 [90.0%]) screened blood for red cell antibodies using a manual method and routine antibody screening and identification was obtainable in only 5 (10.0%) hospitals while extended red cell typing was not available in any hospitals (Table 2). Only 16 (32.0%) centres had facilities for blood component preparation and, of these, 9 (18.0%) and 16 (32.0%) provide cryoprecipitate and platelet concentrate, respectively, while leuco-depleted red cells were not obtainable in any centre (Table 3). Whole blood, packed cell/red cell concentrate and cryoprecipitate account for 57.04%, 28.55% and 2.29%, respectively, of the requests for blood transfusion (Figure 2). The average time to process routine, emergency and uncross-matched requests were a mean of 109.58±79.76 min (range 45.00–360.00), 41.62±25.23 (10.00–240.00) and 11.09±4.92 (2.00–20.00), respectively (Table 4).

**Table 1.** Method of screening for TTIs in hospital-based transfusion in Nigeria

	Routinely screened	
TTI	Yes, n (%)	No, n (%)
HIV	50 (100.00)	0 (0.00)
HBV	50 (100.00)	0 (0.00)
HCV	50 (100.00)	0 (0.00)
Syphilis	40 (80.00)	10 (20.00)
Method of TTIs screening		Frequency (%)
Rapid screening only for HIV, HBV, HCV and syphilis		23 (46.00)
Rapid screening for HIV, HBV, HCV and syphilis then ELISA for only HIV		17 (34.00)
Rapid screening for HIV, HBV, HCV and syphilis then ELISA for all		10 (20.00)
Rapid screening for HIV, HBV, HCV and syphilis then nucleic acid testing for any		0 (0.00)

**Table 2.** Method of blood compatibility testing

Method of blood compatibility testing	Frequency (%)
Manual	45 (90.00)
Semi-automated	3 (6.00)
Fully automated	2 (4.00)
Do you routinely do antibody screening and identification for all patients?	
Yes	5 (10.00)
No	45 (90.00)
Do you routinely do extended red cell typing?	
Yes	0 (0.00)
No	50 (100.00)

**Table 3.** Blood component preparation and availability

Blood component preparation and availability		
Preparation	Yes, n (%)	No, n (%)
Facilities for blood component	16 (32.00)	34 (68.00)
Red cell concentrate	16 (32.00)	34 (68.00)
Leucodepleted red cells	0 (100.00)	50 (0.00)
Irradiated red cells	0 (100.00)	50 (0.00)
Washed red cells	3 (6.00)	47 (94.00)
Fresh frozen plasma	16 (32.00)	34 (68.00)
Cryoprecipitate	9 (18.00)	43 (82.00)
Platelet concentrate	16 (32.00)	34 (68.00)

**Table 4.** Time taken to process blood requests

Time taken to process blood request (minutes)		
Type of request	Mean±SD	Range
Routine request	109.58±79.76	45.00–360.00
Emergency request	41.62±25.23	15.00–240.00
Uncross-matched request	11.09±4.92	5.00–20.00

## Discussion

Blood as a lifesaving tissue should be readily available to all persons in need, with the utmost concern for its adequacy and safety as enshrined in the World Health Assembly resolutions WHA28.72 (1) of 1975 and WHA58.13 (2) of 2005.^14^ Adequate and safe blood depends on the soundness of the blood donation process adopted by the NBTC or blood banks. The low use of donor questionnaires and enrolment forms as well as the high rate of unsigned consent before donation recorded in this study could collectively undermine the safety of blood in the country and cause legal issues in the operation of blood transfusion services. These run counter to the standard of administering questionnaires, completion of enrolment forms and obtaining consent for risk stratification of prospective donors and legitimate protection of the process.^15^ A study by Salamat^16^ in Pakistan reported that appropriate application of a donor questionnaire is important in order to prevent unnecessary blood donor deferral and ensure blood safety. A previous study also reported the use of informed consent in transfusion medicine to be low compared with other procedures in medical practice.^17^

The absence of a donor clinic in most centres and a lack of facilities to establish and nurture donor mobilization and external blood donation drives will hinder the realization of a global framework for action to achieve 100% voluntary blood donation, which ultimately aims to avoid family replacement and paid donations.^18^ The present situation of donor clinics and external donor drives in the participating THs may be partly responsible for the findings of low voluntary non-remunerated (VNR) donors and higher family replacement donors as well as a significant proportion of commercial donors reported by this study. Our finding on blood donation in the country is an improvement from the situation in the recent past when blood was almost exclusively sourced from family replacement and paid commercial donors with negligible or non-existent contributions by VNR donors.^18–21^ Similar, but nonetheless variable, improvement in the contribution of VNR donors was recently noticed in many countries across sub-Saharan Africa (SSA).^22,23^ Factors hindering voluntary donation in SSA were succinctly highlighted by Ugwu et al.,^24^ including taboos, superstitious beliefs, low number of VNR donors and a predominance of family replacement donors. Another challenge that can affect voluntary donation in the hospital setting is the issue of a service fee charged for blood processing. These notwithstanding, attainment of 100% voluntary blood donation is vital to the safety of blood, especially in our setting where a high prevalence of TTIs, poverty and unemployment make any other source of blood extremely dangerous, as prospective donors in the setting of family replacement or paid donations could conceal information on their risk to enable them to fulfil donation criteria either for financial gain or family interest. The commitment of policymakers is the greatest step in transfusion safety, as weaknesses in the regulatory framework, governance and leadership constitute major impediments to transfusion safety in poor economies.^25^

All hospitals were in compliance with World Health Organization (WHO) recommendation of mandatory TTI screening for HIV, HBV and HCV, but for syphilis the compliance was not total. However, the technology for TTI screening remains a serious concern in the safety of blood in Nigeria, as none of the THs employed nucleic acid testing. Nucleic acid, being part of the infectious agent itself, is the first detectable marker of infection to appear before both antigens and antibodies, which form the basis of immunoassays for rapid screening and enzyme-linked immunosorbent assay (ELISA).^14,26–28^ Thus the current methods of TTI screening in Nigeria have extended the window of infectious agents by an estimated 11 to 22 days for HIV, 25–30 to 59 days for HBV and 12 to 70 days for HCV.^26^ This lengthy window period inherent in the current testing methodologies, together with non-use of donor questionnaires and a low proportion of VNRs reported by this study can collectively increase the chance of enrolling/recruiting non-eligible donors and further jeopardize the safety of blood. This concern is paramount in the setting with a high prevalence of TTIs, as there could be significant numbers of window-period donations that can be identified by nucleic acid testing.^14^

The capacity to separate blood to its components across the THs falls short of the 37% reported by the WHO for low-income countries.^18^ This lack of component preparation in most centres restricts the availability of blood components such as red cell concentrate, platelet concentrate, washed red cells, fresh frozen plasma and cryoprecipitate, as well as a total absence of leucodepleted and irradiated red cells in any of the THs. A previous study reported the low availability of blood components with consequent blood wastage.^29^ This same issue of capacity to produce blood components may be responsible for the blood requisition pattern reported in this study, which was majorly whole blood. All these could lead to inappropriate transfusions and wastage of blood and exposure of recipients to the preventable side effects of unwanted components. The chances of exposing a blood recipient to a preventable unwanted effect of blood is further confounded by the method of compatibility testing and the lack of extended red cell typing in all THs. Although some authorities favour transfusion of whole blood over product and component therapy at present in SSA, owing to the peculiar challenges in the region, which includes the rate of emergency transfusion, cost of product preparations, delays in preparation and utility of the plasma for industrial use in view of stringent regulations guiding such among other things.^14,30^

### Conclusions

Hospital-based blood transfusion services in Nigeria depend on family replacement donors who were mostly enrolled without the use of donor questionnaires or were tested for TTIs using a rapid screening method alone. Blood is regularly transfused in whole without blood component therapy and is always processed using manual serological techniques without routine antibody identification or extended red cell typing. The time to process both routine, emergency and uncross-matched blood remain longer than necessary in most THs. These findings are a wake-up call for authorities to provide facilities for blood product preparation and upgrade serological techniques through budgetary allocations and partnerships and for reorientation of physicians and blood transfusion personnel in the use of blood components and prompt response through continuous education and training.

A limitation of the study was the low response rate from many hospitals, which may have affected the results.

## Data Availability

All data generated and analysed during this study are included in this study.
